# Type 2 diabetes, but not obesity, prevalence is positively associated with ambient temperature

**DOI:** 10.1038/srep30409

**Published:** 2016-08-01

**Authors:** John R. Speakman, Sahar Heidari-Bakavoli

**Affiliations:** 1 Institute of Genetics and Developmental Biology, Chinese Academy of Sciences Chaoyang, Beijing 100101 Peoples Republic of China; 2 Institute of Biological and Environmental Science, University of Aberdeen, Aberdeen, Scotland, AB24 2TZ UK; 3 Department of Biology, Faculty of Sciences, Ferdowsi University of Mashhad, Mashhad, Iran

## Abstract

Cold exposure stimulates energy expenditure and glucose disposal. If these factors play a significant role in whole body energy balance, and glucose homeostasis, it is predicted that both obesity and type 2 diabetes prevalence would be lower where it is colder. Previous studies have noted connections between ambient temperature and obesity, but the direction of the effect is confused. No previous studies have explored the link of type 2 diabetes to ambient temperature. We used county level data for obesity and diabetes prevalence across the mainland USA and matched this to county level ambient temperature data. Average ambient temperature explained 5.7% of the spatial variation in obesity and 29.6% of the spatial variation in type 2 diabetes prevalence. Correcting the type 2 diabetes data for the effect of obesity reduced the explained variation to 26.8%. Even when correcting for obesity, poverty and race, ambient temperature explained 12.4% of the variation in the prevalence of type 2 diabetes, and this significant effect remained when latitude was entered into the model as a predictor. When obesity prevalence was corrected for poverty and race the significant effect of temperature disappeared. Enhancing energy expenditure by cold exposure will likely not impact obesity significantly, but may be useful to combat type 2 diabetes.

Obesity and its metabolic sequalae, such as type 2 diabetes mellitus, represent the largest health threat facing the modern world^1^, imposing major economic burdens on healthcare systems and the economy in general^2^. Obesity is defined as excessive storage of lipids, which occurs primarily in white adipose tissue (WAT) depots. It is widely agreed that obesity results from an excess of energy intake over energy expenditure^3^. Energy expenditure is a composite variable comprising the energy expended while resting, physical activity energy expenditure and the thermic effect of food^3^. One factor that is generally considered of negligible importance in the energy budgets of humans is the cost of thermoregulation. This is because it is assumed that humans spend most of their lives at thermoneutral temperatures^4^ where there are no thermoregulatory demands. Yet there is some indirect evidence suggesting that humans do expend energy on thermoregulation.

In addition to white adipose tissue, a second form of adipose tissue occurs, known as brown adipose tissue (BAT)^5^. BAT differs from WAT in that its cells contain multi-locular fat droplets and it is rich in mitochondria^5^ which contain a protein (uncoupling protein 1: UCP1)^5^,^6^ that provides a channel by which protons can bypass ATP synthase, and thereby dissipate their chemiosmotic potential directly as heat^5^,^6^. BAT therefore acts as a thermogenic tissue. It was originally thought that BAT was restricted in its phylogenetic distribution to small mammals and the neonates of larger species^5^, both of which have significant thermoregulatory requirements because of their adverse surface to volume ratios. In recent years however it has been shown that adult humans have functional BAT depots^7^,^8^,^9^,^10^. Previous work has demonstrated that this BAT activity is responsive to changes in ambient temperature^11^,^12^,^13^,^14^,^15^,^16^ and that levels of BAT activity vary seasonally^17^,^18^, being higher in the winter when it is colder. This indirect evidence suggests that humans do indeed expend energy on thermoregulation, and more so when it is colder. This effect occurs despite spending long periods of time indoors buffered from such ambient extremes by the spread of central heating and air-conditioning^19^,^20^.

If these changes have physiological consequences that are not compensated by elevated food intake, then we would predict that the prevalence of obesity, and type 2 diabetes, should be reduced in areas where it is colder. Several previous studies have noted correlations between obesity prevalence and ambient temperatures, or a role for ambient temperature in individual susceptibility to obesity^4^,^21^,^22^, but the results are confused. Some studies have suggested obesity (BMI) is increased at higher temperatures^4^, others have suggested it is decreased^21^, while yet others find no significant effect^22^. The different results between studies may be because there are several important confounding factors, and different studies take them into account to different extents and in different ways. In particular, there are large spatial variations in levels of poverty, which is known to be a key factor linked to the risk of obesity and type 2 diabetes^23^,^24^. Regional or individual differences in poverty levels have not always been taken into account in analyses of temperature effects. Second, there are large spatial variations in the distribution of races that vary in their genetic susceptibility to obesity^25^ and in the conversion probability of obesity to type 2 diabetes^26^. Finally, colder ambient temperatures are generally found in locations at higher latitudes, where sun exposure may be a limiting factor on vitamin D status^27^, which has been implicated as a causal factor in the risk of type 2 diabetes^28^,^29^, although such a link is disputed^30^. Nevertheless a spatial link between ambient temperature and disease prevalence may therefore come about because of these confounding factors. Additionally the role of temperature in individual susceptibilities is made difficult by the problem of ascribing individual temperature exposures. To study the association between ambient temperature and variations in the prevalence of obesity and type 2 diabetes we used the county level disease prevalence data across the mainland USA (downloaded 2014) and matched these to the county level ambient temperature data. Restricting the analysis to spatial variations within a single country (the USA) reduces the potential biases that may occur because of differences in food supply, economic activity and culture. We used the US census data (2010) to control for the potential confounding impacts of poverty and race.

## Results

**Figure 1:**
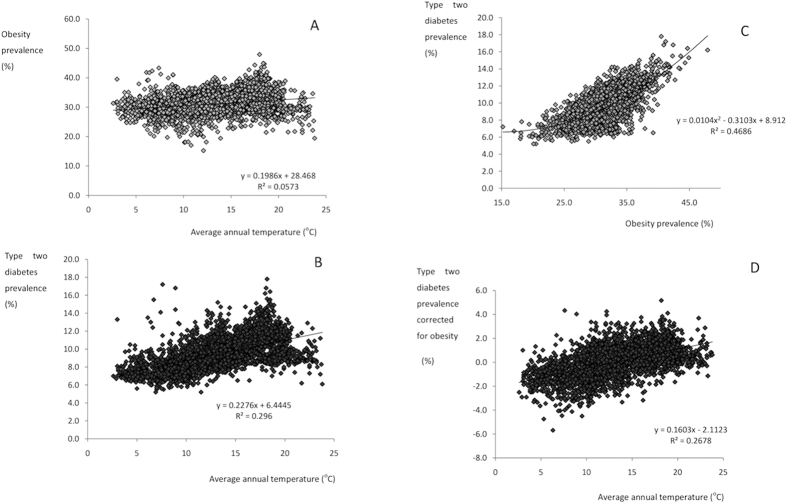
Levels of obesity and type 2 diabetes prevalence across the mainland USA. Plots show the county level data (n = 2655 counties) for (**A**) obesity prevalence (proportion of population with BMI >30) and average annual temperature (^°^C), (**B**) type 2 diabetes prevalence and average annual temperature (^°^C), (**C**) the association between obesity and type 2 diabetes prevalence and (**D**) the association between type 2 diabetes prevalence corrected for obesity levels and average annual temperature (^°^C). Fitted lines show the least squares fit regression equations with associated equations and r^2^ values.

Using the raw unadjusted data there was a significant positive relationship between the prevalence of obesity and ambient temperature (Fig. 1A). The least squares fit regression equation obesity prevalence = 0.1986*(average annual ambient temperature ^°^C) +28.46, explained 5.73% of the variation in obesity prevalence (F_1,2653_ = 161.25, P < 0.0005). The difference between the predicted level of obesity (% population obese) at a mean ambient temperature of 5 °C (29.5%) and 25 °C (33.4%) was approximately 3.9%. Surprisingly the impact of ambient temperature on type 2 diabetes prevalence was considerably stronger (Fig. 1B). The least squares fit regression equation diabetes prevalence = 0.2276*(average annual ambient temperature) +6.44 (Fig. 1B) explained 29.6% of the variation in diabetes prevalence (F_1,2653_ = 1115.26, P < 0.0005). At 5 °C the predicted prevalence was 7.58% compared to 12.13% at 25 °C, about 1.6x greater. This difference in prevalence probably has enormous impacts on healthcare systems in the respective counties. As anticipated there was a strong association between prevalence of obesity and prevalence of type 2 diabetes (r^2^ = 0.468) (Fig. 1C). When we corrected the prevalence of type 2 diabetes for the prevalence of obesity the average annual ambient temperature still explained 26.7% of the residual variation in type 2 diabetes prevalence (Fig. 1D) (F_1,2653_ = 969.53, p < 0.0005).

**Figure 2:**
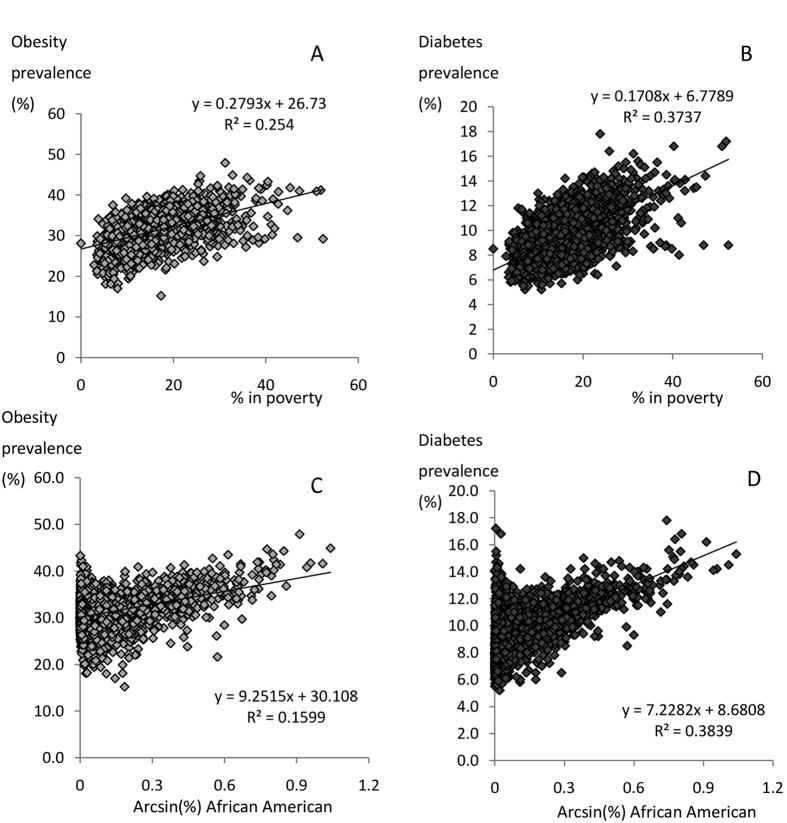
Associations between obesity and type 2 diabetes with poverty and race. Plots show the county level data across the USA (n = 2655) for (**A**) obesity prevalence (proportion of population with BMI >30) and poverty (% of population below poverty line), (**B**) type 2 diabetes prevalence and poverty, (**C**) obesity and the proportion of the population that are African American (arcsin transformed) and (**D**) the association between type 2 diabetes prevalence and the proportion of the population that are African American (arcsin transformed). Fitted lines show the least squares fit regression equations with associated equations and r^2^ values.

**Figure 3:**
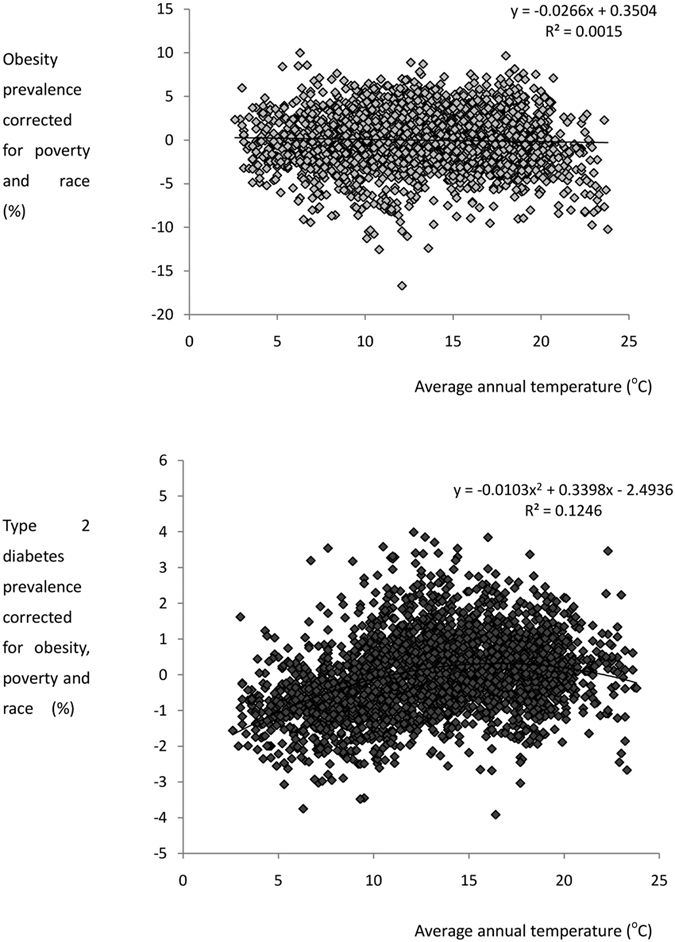
Temperature effect on Type 2 diabetes prevalence across the mainland USA corrected for levels of poverty, obesity and population racial make-up. Plot shows the county level data (n = 2651 counties) for type 2 diabetes corrected for levels of obesity, poverty and race against average annual temperature (^°^C). The curve shows the best fit polynomial regression.

Spatial patterns in both poverty and race mirror the spatial patterns in the levels of obesity and type 2 diabetes. Hence there were strong relationships between both obesity, and type 2 diabetes, prevalence with poverty (Fig. 2A,B). Very similar relationships to those observed with % individuals living in poverty were obtained when using the average county income as the independent predictor. Associations were also noted between obesity and type 2 diabetes with the % of the population in a given area that was African American (Fig. 2C,D). The distributions of these latter race data show heteroscedasticity in the variance. We tried various transformations to remove this but none were completely successful. Fortunately, the outcome of the final analysis was robust to the type of data transformation used. When we corrected the levels of obesity for the levels of both poverty and population racial make-up, the variation in obesity levels explained by ambient temperature fell to 0.15% (F_1,2649_ = 3.85, p = 0.052, Fig. 3a).

**Figure 4:**
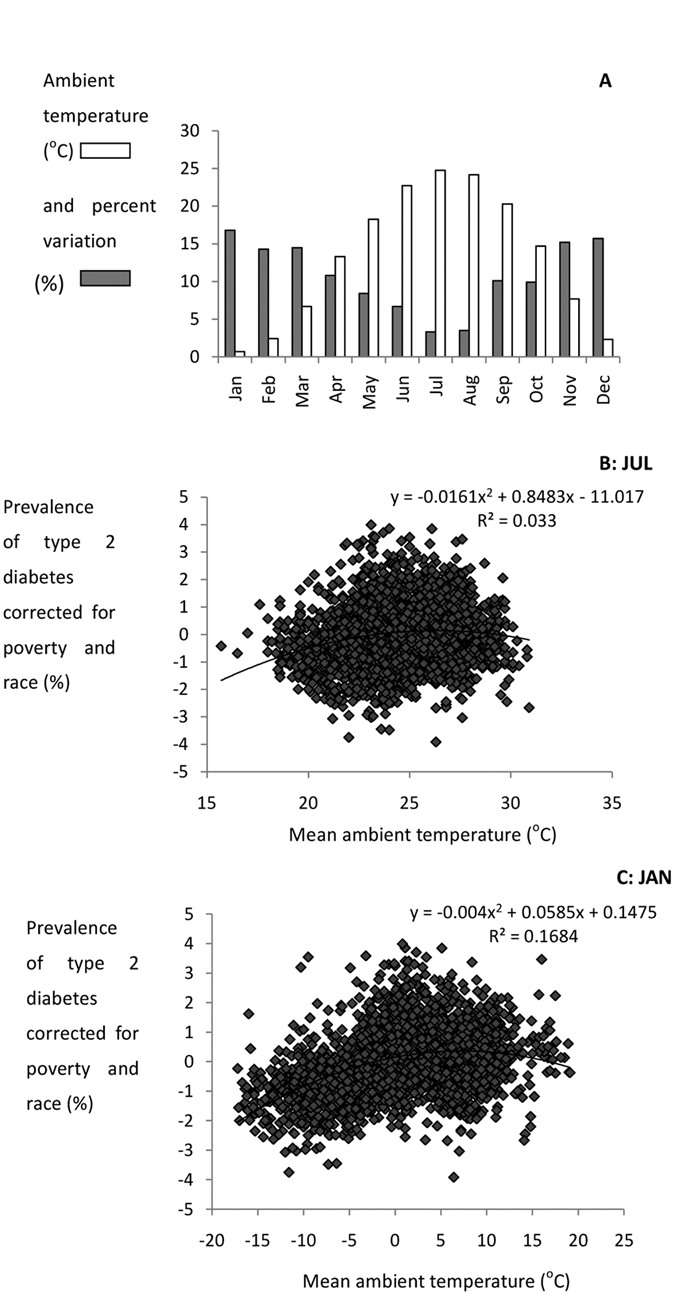
Effects of temperatures in different months on type 2 diabetes prevalence. **(A)** Histogram showing the percentage variation in type 2 diabetes prevalence (corrected for obesity, poverty and race) explained by ambient temperature and temperature squared (gray bars) in each month of the year and the average monthly temperature across all sites (n = 2651) (open bars). The explained variation was greater in months when it was colder. (**B,C**) Example relationships between type 2 diabetes prevalence (corrected for obesity, poverty and race) and ambient temperature in July and January. The curves show the best fit relationships and the associated equations.

In contrast the relationship between the prevalence of type 2 diabetes and ambient temperature when corrected for obesity levels, poverty and race was best fitted by a curvilinear fit that explained 12.5% of the variance in diabetes prevalence (F_2,2648_ = 188.5, p < 0.0005: Fig. 3b). Using the temperature data for individual months, rather than the annual average revealed a systematic change in the explained variation with time of year (Fig. 4a). The explained variation was inversely related to the mean monthly temperature averaged across all the sites. Hence, in mid-summer when the average temperature was 24.8 °C (July) the explained variation in type 2 diabetes prevalence (corrected for poverty, obesity and race) by ambient temperature and temperature squared was only 3.3% (Fig. 4b). In contrast in mid-winter when the average temperature across all sites was 0.7 °C (January) the explained variation in type 2 diabetes prevalence by the same parameters was 16.8% (Fig. 4c). In part this was because in winter the range of temperatures was also greater (Fig. 4). When we included latitude as a predictor in the model it explained additional variance to the temperature effect and both temperature, temperature^2^ and latitude were highly significant independent predictors (e.g. for the January temperature effect the explained variance by temperature and temperature^2^ was 16.8% (F_2,2648_ = 268.0, p < 0.0005) and when latitude was included the explained variation increased to 20.1% (F_3,2647_ = 221.98, p < 0.0005).

## Discussion

The absence of an ambient temperature effect on obesity levels could occur for several reasons. For example, individuals may spend most of their time indoors and are simply not exposed to the external ambient temperature conditions for long enough to have an impact on energy balance. Although historically indoor temperatures may have been closely correlated with external temperatures there has been a spread in central heating and air conditioning over the last 5 decades^19^,^20^ which has also served to homogenize the home temperature experience independent of the external ambient conditions. This interpretation however is inconsistent with the observation that levels of BAT activity vary seasonally^17^,^18^, being higher in the winter when it is colder. This suggests then that the known activation of brown adipose tissue at colder temperatures does not impact on whole body energy balance or weight regulation. This could for example be because any elevation in energy expenditure is offset by a compensatory increase in appetite and food intake^3^. These data are consistent with a previous study of impacts of ambient temperature on obesity prevalence^22^ but contrast other studies where effects have been observed^4^,^21^. These latter observations may be because of inadequate control for confounding factors or using different measures of temperature.

Our analysis suggests that while ambient temperature did not have a major impact on the levels of obesity it did have an impact on the prevalence of type 2 diabetes explaining up to 16.8% of the variation in diabetes levels between counties. Although reduced when compared to the uncorrected prevalence data, 16.8% remains a substantial level of explained variance. To set this finding in context, this effect of ambient temperature on type 2 diabetes prevalence is greater than the total variation explained from the combined effects of all the genetic polymorphisms previously identified by genome wide association studies for type 2 diabetes which stands at around 10% of the variation^31^. Although we have called this type 2 diabetes, given the nature of the survey data (see methods) a small percentage of the adult diabetic population will have type 1 diabetes. The trends we describe are unlikely to be a consequence of differences in the prevalence of type 1 diabetes because the county wide differences exceed the estimated type 1 diabetic proportion. Moreover, there is a well established increase in the prevalence of pediatric type 1 diabetes at higher latitudes^32^ that has been ascribed to lower temperatures^33^ (i.e. opposite the trends described here for adults).

One potential reason for this effect on type 2 diabetes, in the absence of a link to obesity, might be that the association between prevalence of type 2 diabetes and temperature comes about because of an artefact of an association between both ambient temperature and type 2 diabetes with latitude. Latitude may be of significance for type 2 diabetes susceptibility, but not obesity, because at higher latitudes there is reduced synthesis of vitamin D due to lower levels of sunlight exposure^27^ and vitamin D status has been implicated in the risk of type 2 diabetes^28^,^29^. Two factors suggest this is unlikely. The association between vitamin D status and type 2 diabetes risk, if it is causal^30^, if anything would cause a greater risk at higher latitudes, yet it was at such higher (colder) latitudes where the risk was lowest. Moreover, when we included latitude into the model the temperature effect was not diminished, but latitude instead entered as an additional explanatory factor. This strongly indicates that the temperature effect on type 2 diabetes prevalence was not an artefact of a latitude effect acting via vitamin D status, or any other latitude related factor. Indeed another factor that is strongly related to latitude is susceptibility to mental illness^34^. Since risk of type 2 diabetes is increased in patients with mental illness, one might anticipate that type 2 diabetes prevalence would actually increase at higher latitudes and hence lower temperatures, due to this confound. The fact the opposite trend is observed illustrates the strength of the temperature effect. The trend of diabetes prevalence with ambient temperature possibly explains the previously reported negative association of diabetes with altitude^35^.

The curved relationship between type 2 diabetes prevalence and ambient temperature indicated a relatively smaller effect at higher temperatures and a larger effect when the average temperature fell below 18 °C. This shape of the curve was consistent with the human thermoregulatory response to temperature^36^,^37^,^38^ and was hence consistent with the protective effect of low temperature on type 2 diabetes prevalence being linked to switching on thermoregulatory energy expenditure when it is colder. This interpretation is also consistent with the changing level of explained variation in type 2 diabetes prevalence in different seasons. That is in mid-summer most of the temperature data sit above the lower critical temperature^38^,^39^,^40^ where there is minimal thermoregulatory demand, and in this situation the association between ambient temperature and type 2 diabetes prevalence was lower than in winter when most data sit below this level. Consequently the variation in July temperatures would be unlikely to be related to the annual level of thermoregulatory requirement, while temperatures in January would be strongly linked to such a requirement.

Low temperature may have an impact on the prevalence of type 2 diabetes in the absence of an effect on obesity because colder ambient temperatures stimulate brown adipose tissue^12^,^13^,^14^,^15^,^16^ which is capable of disposing large quantities of glucose and lipids^12^,^39^. The main weakness of such an interpretation is that there is no direct evidence for the US population that BAT is seasonally activated in populations living in colder regions, although this is known to be the case in Japan^11^ and in Europe^18^. It is possible that US populations respond differently to Japanese and European ones in this respect. Interestingly, a small clinical trial recently showed that mild cold exposure (15 °C) for 6 hours per day had marked benefits with respect to glucose homeostasis, and this happened despite only a modest increase in brown adipose tissue activation^40^. Although unfeasible as a clinical treatment option, this points to the possibility that cold may activate other, brown adipose tissue independent, pathways that influence glucose homeostasis, explaining the patterns we observed. If this is true, understanding the impact of cold on glucose homeostasis in humans should become a key future goal.

## Methods

We downloaded the county level data on the age adjusted prevalence of obesity and type 2 diabetes from the USA Centers for Disease Control and prevention web site (www.cdc.gov) (data download July 2014). This website provides data on the current prevalence rates of both diseases across 3146 counties or county equivalents from the continental USA. Excluding counties for which no data were available or for which temperature, poverty and race data were unavailable, left 2654 counties with complete data, comprising a total population of 170,430,015 individuals. The data are sorted by state and by the Federal information processing standard (FIPS) code, which is a 5 digit code that allows counties and county equivalent units to be uniquely identified. Data downloaded for this analysis relate to age adjusted prevalence. Detailed methods for how the county level data are compiled are available on the CDC web site (http://www.cdc.gov/diabetes/pdfs/data/). Briefly the prevalence of diabetes and obesity is estimated using data from the CDC Behavioural Risk factor surveillance system which is a monthly state based telephone survey of a nationally representative sample of adults aged >20 years old. Because it is telephone based it excludes individuals living in care homes or those without a telephone. The survey was changed in 2011 to include cell phone numbers. More than 400,000 individuals are contacted annually to take part in the survey which has been running since 1984. Individuals are judged to have diabetes if they respond ‘yes’ ; to the question “Has a doctor ever told you that you have diabetes”? excluding females who indicate in a follow-up question that they only had diabetes during pregnancy. Previous work indicates that self report of a physician’s prior diagnosis of diabetes is highly reliable compared to medical records^41^. This question does not separate those with type 1 and type 2 diabetes. In the adult population of the USA more than 96% of diabetes is type 2, we therefore called the estimated prevalence that of type 2 diabetes. Given the magnitudes of the trends described here they cannot be attributed to differences in prevalence of the type 1 diabetes. For obesity, in the telephone interview, individuals self report their height and weight in response to the questions “About how much do you weigh without shoes”? and “About how tall are you without shoes”? which are then converted if necessary to kg and metres before calculating the Body mass index (BMI = (height)^2^/weight). A BMI >30 is then classed as obese using the WHO standard for Caucasians^1^. This is applied independent of actual race. Individuals normally over estimate their own height and underestimate their own weight in a self report setting^42^,^43^ and hence these estimates are likely to be conservative. However, we considered it unlikely that individuals would be deceptive about their diabetes status, height and weight in relation to ambient temperature and hence it seems improbable that the reported trends are a consequence of such biases. In addition to the health questions individuals are also asked a core of demographic questions which include age, sex, race, marital status, education, employment status, income and home ownership status.

The individual county level data for the BRFSS using the core demographic data are then imputed to yield county wide level statistics using the US Census bureau 2010 census data. Rates of obesity and diabetes are age adjusted by calculating age specific rates for 3 age groups 20–44, 45–64 and >65. A weighted sum based on the distribution of these age groups from the census is then calculated. The imputation of the specific county level rates of prevalence is based on a Bayesian multilevel modelling approach that utilises data from adjacent counties to refine the predictions. This causes a potential issue because the estimates for each county are potentially not independent of those for adjacent counties. We addressed this issue using a variogram analysis as described below.

We combined the estimated prevalence data for diabetes and obesity with the monthly temperature records for each county (identified also by FIPS code) available from the Oak Ridge National laboratory (http://www.daac.ornl.gov: files B01, B02 and C07), and the records of county level poverty (% in poverty and average income) and racial make-up data from the United States Census Bureau, 2010 census data (http://www.census.gov/2010census specifically files PVY01, PVY02, INC01, INCO2, INCO3, IPE01, RHI02). This allowed us to then normalise the prevalence data at the county level for these confounds and seek associations of temperature to the disease prevalence’s corrected for race and poverty.

**Figure 5:**
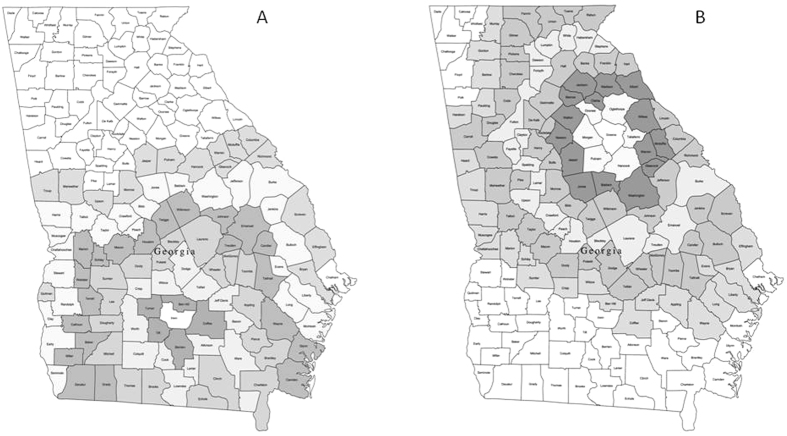
Illustration of selection process for variogram analysis. Shaded map of Georgia state to illustrate the variogram analysis. (**A**) shows the counties surrounding Greene county up to 6 steps away and Fig. 1B shows the same for Irwin county. Original county map purchased from www.mapresources.com.

To establish whether the county level data represent independent sampling units we performed a variogram analysis on the residual type 2 diabetes prevalence accounting for the effects of obesity, poverty and race. We performed this analysis for 3 southern (Alabama, Georgia and Mississippi) and 3 northern (Iowa, Minnesota and North Dakota) states. The variogram analysis involves looking at the correlations between each county and its immediate neighbours, and then the correlation between each county and the neighbours that can only be reached by passing through 1 other county, then 2 other counties away, etc up to 6 steps away from a given start point. To illustrate this analysis Fig. 5A shows the Georgia county map shaded to show the counties in increasing steps around Greene county. We recorded the data for residual diabetes prevalence in Greene county and all the counties surrounding it up to 6 steps away. We then repeated this process using different counties within the state (Fig. 5B for example shows the same county map of Georgia now shaded for Irwin county). If the county is an appropriate level of investigation then there should not be a significant downward trend in the correlation as the number of steps increases indicating that the correlation to the nearest neighbour is no better than the correlation to neighbours 1,2,3 etc. steps away.

**Figure 6:**
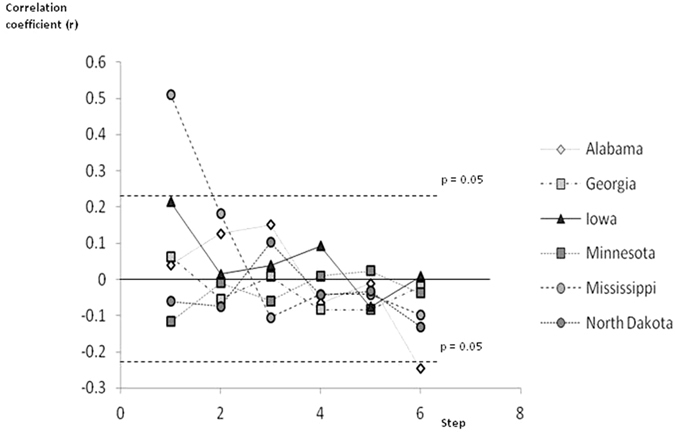
Variogram analysis. The plot shows the correlation in relation to the step away from the focal county for each of the 6 states that were analysed (see Fig. 5). The approximate limits for the 95% significance levels (Bonferoni corrected) are also shown as dashed lines (p = 0.05). Values falling outside these two lines were significant.

The correlation coefficients in relation to step number for each of the 6 states included in the variogram analysis are shown in Fig. 6. In all the cases except 2 the correlation coefficients fell inside the 95% confidence limits (corrected for multiple testing by the Bonferoni method). Actual required p to reach significance = 0.05/36 = 0.0014. The two exceptions were for 6 steps in Alabama where there was a negative correlation and for one step in Mississippi where there was a positive correlation. This positive correlation in Mississippi may suggest that the county levels of residual diabetes prevalence are not entirely independent of those reported in neighboring counties. However, the fact this pattern was only observed in a single state from the six we studied in detail indicates that this phenomenon is unlikely to seriously inflate the significance of the data included into the analysis and that in general the county is an appropriate level at which to conduct spatial analysis of such disease data.

## Additional Information

**How to cite this article**: Speakman, J. R. and Heidari-Bakavoli, S. Type 2 diabetes, but not obesity, prevalence is positively associated with ambient temperature. *Sci. Rep.* **6**, 30409; doi: 10.1038/srep30409 (2016).
